# Editorial: Regenerative Medicine for Cartilage and Joint Repair

**DOI:** 10.3389/fbioe.2022.891970

**Published:** 2022-04-13

**Authors:** Zhen Li, Laura Creemers, Xiaoling Zhang

**Affiliations:** ^1^ AO Research Institute Davos, Davos, Switzerland; ^2^ Department of Orthopaedics, University Medical Center Utrecht, Utrecht, Netherlands; ^3^ School of Medicine, Xinhua Hospital, Shanghai Jiaotong University, Shanghai, China

**Keywords:** cartilage, joint, regenerative medicine, cells, materials

## Introduction

Cartilage diseases affect a large population worldwide and are associated with a significant burden to patients and society. Osteoarthritis (OA) is the most common chronic joint disorder and the fastest growing cause of disability. Cartilage focal defects, predisposing to early OA and degeneration impair the function of many joints, including the articular joint, intervertebral disc and temporomandibular joint. Presently, no effective therapy is available, except for palliative care primarily used to delay invasive and often suboptimal surgical interventions. Regenerative medicine therapies directed at the early stage of osteoarthritis, and tissue engineering approaches to reconstruct cartilage defects provide great potential for cartilage and joint repair in the future. This current research topic represents a collected series of articles in this field. Highlights are summarized below.

## Research Topic Highlights

Liu et al. provided a timely review describing the factors that affect cartilage homeostasis and function, and the emerging regenerative approaches that are informing the future treatment options. Tissue engineering approaches combining cell and biomaterial strategies to reconstruct the complex and functional cartilage tissue are still under active research. Two review articles in the current topic have described the use of 3D printing technology in cartilage tissue reconstruction in general (Perera et al.) and for irregularly shaped cartilage in particular (Wang et al.). Electrospun gelatin/polycaprolactone (GT/PCL) nanofiber membranes at optimum GT/PCL ratio were used to support *in vivo* cartilage regeneration from autologous chondrocytes in a swine model (Zheng et al.). Also xenogeneic acellular cartilage matrix (ACM) materials encapsulated with autologous chondrocytes showed capacity to promote cell proliferation and cartilage formation in goat, with only a minor immune-inflammatory response (Jia et al.). This provides scientific evidence for future clinical application of ACM in cartilage tissue engineering. Next to biomaterials, appropriate cell sources are important for cartilage repair. Combined physioxia and fibronectin adherence have shown to select and propagate a meniscus progenitor population that can potentially be used to treat meniscal tears or defects (Pattappa et al.). Additional stimuli can further enhance the cartilage repair potential of cells. For example, a magnetic field applied to magnetic nanoparticles-loaded cells decreased cellular senescence and enhanced chondrogenic capability of adipose-derived mesenchymal stem cells (Labusca et al.). Senescence can also be targeted using a senolytic peptide, fork head box O transcription factor 4-D-Retro-Inverso (FOXO4-DRI), which removed the senescent cells among chondrocytes (Huang et al.).

Last but not least, appropriate animal models for OA are still lacking and play a pivotal role in the development of regenerative therapies. Bi et al. have established an model combining transection of the anterior and posterior cruciate ligaments, and the meniscus, and a cartilage defect in rhesus macaques, which closely resembled the pathophysiological processes of spontaneous knee OA in humans.

